# Editorial: Role of brain oscillations in neurocognitive control systems

**DOI:** 10.3389/fnsys.2023.1182496

**Published:** 2023-03-31

**Authors:** Golnaz Baghdadi, Chella Kamarajan, Fatemeh Hadaeghi

**Affiliations:** ^1^Department of Biomedical Engineering, Amirkabir University of Technology, Tehran, Iran; ^2^Department of Psychiatry, Downstate Health Sciences University, Brooklyn, NY, United States; ^3^Institute for Computational Neuroscience, Center for Experimental Medicine, University Medical Center Hamburg-Eppendorf (UKE), Hamburg, Germany

**Keywords:** brain oscillations, cognitive systems, neural networks, neural synchronization, neuromodulation, machine learning, EEG, motor control system

Neural activities as reflected in brain oscillations are the “building blocks” of neurocognitive functions (Basar and Guntekin, 2009; Basar, 2013). There is evidence to suggest that spiking neurons can be described as oscillators (Stiefel and Ermentrout, 2016) and cognitive functions are shaped and regulated by rhythmic or chaotic oscillations of these oscillators (Babloyantz, 1987; Kahana, 2006; Lin et al., 2021). Although the exact roles of oscillatory activities remain unclear, results of animal and human laboratory research and simulations of computational models have suggested several hypotheses and theories about the role and mechanism of neuronal oscillatory patterns in brain functions.

As a prominent example of such neurophysiologically-grounded theories, adaptive resonance theory (ART) provides a comprehensive framework for understanding how different cognitive functions are formed through the synchronization of neuronal oscillations (Carpenter and Grossberg, 1998; Grossberg, 2013, 2021a,b,c). ART proposes that the brain consists of networks of neurons that interact to form resonant states, which selectively attend to relevant information (Grossberg, 2013). The synchronization of neuronal oscillations in different frequency bands is a critical factor in the formation of resonant states, according to ART. Additionally, this theory proposes that resonance is a fundamental mechanism for the formation and maintenance of memories. This theory has received empirical support and is widely recognized as an influential theory in the field of cognitive neuroscience.

Communication through coherence (CTC) is another widely accepted theory that explains how phase synchronization of brain oscillations facilitates communication and information transmission between different brain regions (Fries, 2015; Gonzalez et al., 2020; Pérez-Cervera et al., 2020). While numerous studies have explored the synchrony between neuronal oscillations, recent research has also shown that the top-down attention system can attenuate the effects of distractors by sending signals that cause desynchronization of neuronal oscillations (Baghdadi et al., 2019). This highlights the dynamic and adaptable nature of brain oscillations and their role in regulating cognitive processes. Additionally, neurophysiological recordings have shown that brain oscillations play a role in regulating the timing of neuronal spiking, which supports the temporal-coding hypothesis (Jacobs et al., 2007; Singer, 2018). Brain oscillations are also associated with the organization of different categories of information and the formation of schemas, further underscoring their critical role in the regulation and coordination of cognitive processes (Ohki and Takei, 2018).

In general, the brain is considered an orchestra hall, during which we see purposeful coordination between brain oscillations (Mazaheri et al., 2018), and this coordination is controlled and adjusted under the influence of important parameters involved in each task (Kahana, 2006; Basar, 2013; Cebolla and Cheron, 2019). However, the neuronal basis and mechanisms of how brain oscillations are regulated by the features of different cognitive tasks or how different oscillatory frequencies create brain functions remain largely unknown and there is a need for more studies and experiments, which are discussed in this Research Topic.

In this Research Topic, Ebrahimzadeh, Saharkhiz et al. reviewed the application of simultaneous assessment of electroencephalogram (EEG) and functional magnetic resonance imaging (fMRI) as an effective method for characterizing the roles of brain oscillations in terms of both network topology and the neurophysiological mechanisms. The authors also provide a comprehensive review of research findings related to the involvement of brain oscillations in various cognitive functions (e.g., learning, thinking, reasoning, remembering, problem solving, decision making, and attention) and neurocognitive disorders (e.g., post-traumatic stress disorder, Alzheimer's disease, epilepsy, and sleep disorders).

Moenne-Loccoz et al. investigated the association between neural oscillatory activity in the motor cortex and striatum and varying degrees of motor activity under normal and parkinsonian conditions, and demonstrated the modulatory effects of local field potential (LFP) oscillations on the dopamine level in rats (normal and model of Parkinson's disease). Changes in broad-band oscillatory activities of cortico-basal ganglia networks (including changes in the relative power of low- and high-frequency bands) were correlated to ongoing motions, reflecting that these oscillatory patterns are modulated by the neural motor control system.

Moghadam et al. developed a computational model of navigation focusing on mutual interactions between the hippocampus (HPC) and medial prefrontal cortex (mPFC). Results of the model simulations indicated that changes in the frequency characteristics of neuronal oscillations can impair the recall and retrieval of required information in navigation, leading to spatial disorientation in Alzheimer's patients.

As mentioned before, CTC is a theory that emphasizes the role of brain oscillations in connecting and communicating between neural networks. Soleimani et al. demonstrated that transcranial direct current stimulation (tDCS) can modulate large-scale neural connectivity. In a study investigating the neural circuitry underlying methamphetamine use disorder, the authors found that bilateral tDCS can increase cortical excitability in the executive control network (ECN) and ventral attention network (VAN) networks while having opposite effects on the default mode network (DMN). This research highlights the potential for externally induced stimulation methods like tDCS to modulate brain oscillations.

Finally, Ebrahimzadeh, Fayaz et al. have demonstrated the utility of machine learning approach for classifying patients with major depression disorder (MDD) who responded to repetitive transcranial magnetic stimulation (rTMS) treatment from the non-responders using resting state EEG data. K-nearest neighbor (KNN), support vector machine (SVM), and multilayer perceptron (MLP) algorithms classified the responders vs. non-responders using relevant features extracted from the EEG time series. The strongest discriminative indicators were EEG beta power, the sum of bispectrum diagonal elements in delta and beta bands, and correlation dimension. This study suggests that EEG assessments have the potential to predict the effectiveness of rTMS as a treatment for patients with MDD.

Building on the previous Research Topic on “*Why the exact frequencies in our brains matter*” (Hadjipapas et al., 2022), the current Research Topic reveals that brain oscillations have significant implications for multiple aspects of neural system function. As such, investigating the underlying mechanisms of brain oscillation modulation could potentially lead to the development or optimization of therapeutic or rehabilitative techniques. To this end, Ebrahimzadeh, Fayaz et al. and Moenne-Loccoz et al. have highlighted the value of utilizing brain oscillatory features to explore various disorders, which may potentially guide neuromodulatory interventions, including repetitive transcranial magnetic stimulation (rTMS).

As an increasing number of studies investigate the contribution of brain oscillations to neurocognitive control systems, the articles featured in this Research Topic demonstrate the value of using neural oscillations to investigate both healthy and pathological brain states as well as to assess treatment outcomes of neuromodulation methods such as rTMS. We believe that brain oscillatory measures will continue to serve as critical non-invasive tools for comprehensively understanding neurocognitive systems, given their high sensitivity and accuracy.

## Author contributions

All authors listed have made a substantial, direct, and intellectual contribution to the work and approved it for publication.
